# The Causal Relationship Between Rheumatoid Arthritis and Mechanical Complications of Prosthesis After Arthroplasty: A Two-Sample Mendelian Randomization Study

**DOI:** 10.3389/fgene.2022.822448

**Published:** 2022-04-05

**Authors:** Yuanqing Cai, Guangyang Zhang, Jialin Liang, Zhaopu Jing, Rupeng Zhang, Leifeng Lv, Xiaoqian Dang

**Affiliations:** Department of Orthopaedics, the Second Affiliated Hospital of Xi’an Jiaotong University, Xi’an, China

**Keywords:** rheumatoid arthritis, mechanical complications, Mendelian randomization, arthroplasty, study

## Abstract

The causal effects of rheumatoid arthritis (RA) on complications of arthroplasty are yet to be established. This study was the first to explore the causal effect of RA on mechanical complications of prosthesis through two-sample Mendelian randomization (MR). In the MR analysis, RA was selected as the exposure in this study while single-nucleotide polymorphisms (SNPs) from a genome-wide association study (GWAS) were selected as the instrumental variables (IVs). Summary statistics data on mechanical complications of prosthesis was extracted from publicly available GWAS data, including 463,010 European descent individuals. MR analysis was performed using the standard inverse variance weighted method (IVW). Furthermore, other methods (MR Egger, weighted median, simple mode, and weighted mode) were also done to verify the results. Finally, the sensitivity analysis was executed. Results of the standard IVW showed that RA possibly increases the risk of mechanical complications of prosthesis [OR = 1.000255; 95% CI = (1.0001035, 1.000406); *p* = 9.69 × 10^**−4**^]. This outcome was also verified by other methods including weighted median [OR = 1.000285; 95% CI = (1.0001032, 1.000466); *p* = 1.41 × 10^−3^], simple mode [OR = 1.000446; 95% CI = (1.0001116, 1.000781); *p* = 1.04 × 10^−2^], and weighted mode [OR = 1.000285; 95% CI = (1.0001032, 1.000466); *p* = 2.29 × 10^−3^]. No heterogeneity and directional pleiotropy was observed upon sensitivity analysis, indicating the stability and reliability of the result. In summary, the present study showed that RA potentially increases the risks of complications of prosthesis, which might provide guidance in arthroplasty on RA patients.

## Introduction

Arthroplasty is a common surgical strategy in the management of end-stage arthritis, with the aim of restoring joint function and improving the patient’s quality of life (Lands et al., 2021; Mora and GilesScuderi 2020). However, postoperative complications including periprosthetic fracture, periprosthetic osteolysis, periprosthetic joint infections, and aseptic prosthesis loosening (Chapman et al., 2020; Li, Ma, and Xiao 2019; Renard et al., 2020; Rojas et al., 2019) are inevitable. These greatly reduce patient’s satisfaction, causing significant hindrance in the development of arthroplasty. On the other hand, postoperative mechanical complications of prosthesis including dislocation, wearing, and prosthesis fracture (Brown, Engh, and Fricka 2019; Chakravarty et al., 2015; Rowan et al., 2018) are common, which might eventually lead to arthroplasty failure. Due to the growing number of patients undergoing arthroplasty for various joint diseases (Pollock et al., 2016), identification of factors associated with arthroplasty complications, especially mechanical complications, is of great importance. This has the potential to deeply improve our understanding and greatly reduce the incidence of arthroplasty complications while ensuring good clinical outcomes.

Rheumatoid arthritis (RA) is a chronic systemic autoimmune disease characterized by joint synovitis (McInnes and Schett 2011; Wasserman 2011). RA potentially results in the deformation of joints, destruction of articular tissues, and permanent loss of joint function. Arthroplasty is the last resort to recover the joint function in RA patients (Cho, Kim, and Song 2017; Fan et al., 2019). Presently, the pathogenesis of RA is yet to be completely elucidated. Although both genetic and environmental factors have a potential role in this process, immune dysfunction is considered as the main pathogenesis up to date (McInnes and Schett, 2011). Since the pathogenesis of RA is different from other articular diseases such as osteoarthritis and osteonecrosis, the outcomes and rates of complications after arthroplasty of RA might differ from that of other articular diseases (Blevins and Jason. 2019; Goodman 2015; Goodman et al., 2020). For example, systemic inflammation potentially promotes local osteolysis mediated by articular inflammation, resulting in an increased rate of aseptic loosening (Böhler et al., 2020). Furthermore, previous studies also demonstrated the possible relationship between RA and a higher rate of postoperative complications such as disability and venous thromboembolisms (Pedersen et al., 2014; Vakharia et al., 2020). At present, the correlation between RA and arthroplasty complications is yet to be fully clarified and considered controversial as most studies are retrospective (Blevins and Jason. 2019; Böhler et al., 2020; Grzelecki et al., 2019; Pedersen et al., 2014; Vakharia et al., 2020). The retrospective nature of these studies fails to eliminate bias and confounders, which might lead to false causality.

Mendelian randomization (MR) is a novel approach in which genetic variants serve as instrumental variables to determine the causal effect of exposure on the outcome. The observational research results were unable to provide strong evidence for the causal effects and the formulation of prevention and control strategies secondary to confounders and biases (Talari and Goyal 2020). On the other hand, randomized controlled trial (RCT) is the golden standard for causality evaluation, but execution of a RCT with a large sample size is difficult due to limitations in cost and time (Serra-Aracil et al., 2020). Based on the above concerns, the MR proposed by Katan (1986) might be an ideal choice, which can potentially overcome some of the limitations inherent in traditional epidemiologic studies. MR has become a promising research tool in recent years as it is not affected by common confounding factors and the causal sequence is reasonable and has unique advantages for judging causal inference between exposure factors and outcomes. In addition, MR has been applied in many studies to estimate causal relationship (Qu et al., 2020; Qu and Yang., 2021; Wu et al., 2020; Yeung and Mary Schooling 2021).

The aim of this study was to explore the causal effect of RA on arthroplasty complications, specifically mechanical complications, through two-sample Mendelian randomization. To the best of the author’s knowledge, this was the first study to explore the causal effect of RA on mechanical complications of arthroplasty through Mendelian randomization.

## Materials and Methods

### Study Design

**FIGURE 1:**
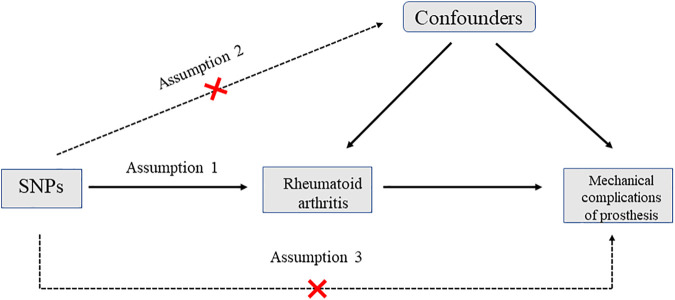
Diagram for Mendelian randomization (MR). There are three assumptions that should be met in MR analysis: first, SNPs should be strongly associated with the exposure; second, selected SNPs should be independent of confounders; third, SNPs should affect the outcome only by the exposure rather than *via* a direct correlation. SNP: single nucleotide polymorphism.

In the present study, two-sample MR was used to evaluate the causal effect of RA on mechanical complications of prosthesis. Any type of genetic variation can be regarded as instrumental variables (IVs) in MR analysis. In this study, single nucleotide polymorphism (SNP) was selected as the IV. Three conditions had to be fulfilled for MR analysis: first, SNPs which were strongly associated with RA (exposure) must be strictly screened and weak IVs have to be removed according to F-static; second, selected SNPs must be independent of confounders. It was verified using the pleiotropy test; third, SNPs must affect mechanical complications of prosthesis (outcome) only by RA (exposure) rather than *via* a direct correlation. It was evaluated by using many MR methods (Figure 1).

### Data Source and Instrumental Variable Selection

This two-sample MR study was performed based on GWAS summary data. In addition, both the GWAS summary data were based on European ancestry. RA summary statistics data published by Stahl et al.,(2010), including 5,539 auto-antibody positive RA cases and 20,169 controls of European descent, were identified through a GWAS meta-analysis and applied in this study (Sample 1). RA was chosen as the exposure variable in this study. The definition of RA was based on the 1987 American College of Rheumatology (ACR) criteria for diagnosis of RA50 or diagnosed by board-certified rheumatologists. Further limitation to anti-CCP+ or RF+ patients in the setting unavailable of anti-CCP status data was done. Genome-wide significant (*p* < 5 × 10^−8^) SNPs were extracted as instrumental variables, and linkage disequilibrium was tested (*r* ^2^ < 0.1). Harmonizing was conducted to ensure that the effect allele of IVs in the exposure and outcome are consistent across different databases. To avoid the reverse causality in MR analysis, the Steiger filtering test was also performed to exclude SNPs that explain more of the variance in the outcome rather than the exposure.

### Genetic Associations With Mechanical Complication of Prosthesis

For the outcome data, summary statistics for mechanical complications of prosthesis were obtained from a recent, publicly available GWAS data (https://gwas.mrcieu.ac.uk/datasets). The data had a total of 463,010 European descent individuals, including 1,178 cases and 461,832 controls of European descent. The mechanical complication of internal joint prosthesis conformed to the main ICD10: T84.0 diagnosis criteria. All studies that contributed data in the analyses were approved by relevant ethics committees, and all participants have provided written informed consent.

### Statistical Analysis

SNPs with F-statistic ≥ 10 were selected for further analysis. Primary analysis of the causal effect of RA on mechanical complication of prosthesis was performed using the standard inverse variance weighted (IVW) method (Luo et al., 2020). The Wald ratio of each SNP was calculated to assess the causal effects of each SNP on the outcome. Finally, the inverse variances of SNPs were utilized as weight for meta-analysis to evaluate the combined causal effect.

Furthermore, other MR methods, including MR Egger, weighted median, simple mode, and weighted mode, were also used to estimate the causal effect of RA on mechanical complications of prosthesis (Bowden, et al., 2016b; Bowden, et al., 2016a).

Considering that biases might be caused by IVW in the presence of horizontal pleiotropy, sensitivity analysis, including the heterogeneity test, pleiotropy test, and leave-one-out sensitivity test, was also performed to assess the reliability and the stability of MR results. In addition, the MR-PRESSO (Mendelian Randomization Pleiotropy RESidual Sum and Outlier) test was conducted to detect potential outliers and pleiotropy (Verbanck et al., 2018). The intercept of the MR-egger further tests the horizontal pleiotropy (*p* < 0.05). A web-based application (https://sb452.shinyapps.io/power/) was used to calculate the power of the MR analysis.

All statistical analysis were performed in R software (Version 3.6.1) with the R package “TwosampleMR.” The calculations are available in Supplementary Material S1, with *p* < 0.05 considered to be statistically significant. Since publicly available summary data was used in this study, no ethical approval was needed.

## Results

### Selection of Instrumental Variables

To assess the causal effect of RA on mechanical complications of prosthesis, the significant (*p* < 5 × 10^−8^) and independent (*r* ^2^ < 0.1) SNPs were selected for MR analysis. A total of 115 SNPs were found to be significantly associated with RA. Twenty-six SNPs were selected as IVs after harmonising and steiger filtering for further MR analysis with a resulting mean F-statistic of 193.16 and average R2 of 0.007. The details of these SNPs are provided in Supplementary Material S2.

### Causal Effect of RA on Mechanical Complications of Prosthesis

**FIGURE 2:**
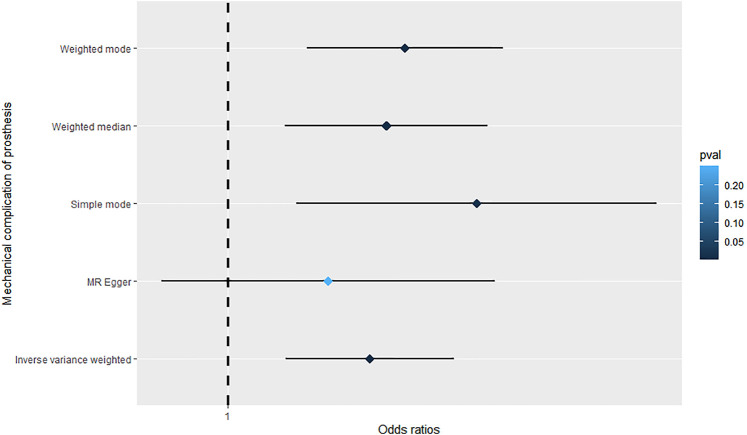
Forrest plot of the causal effects of RA-associated SNPs on mechanical complications of prosthesis.

The MR analysis results are summarized in Table 1; Figure 2. As shown in the table and figure, the causal effect of RA on mechanical complications of prosthesis analyzed by using the IVW method suggests a positive effect [OR = 1.000255; 95% CI = (1.0001035, 1.000406); *p* = 9.69 × 10^−4^]. Further MR analysis using weighted median [OR = 1.000285; 95% CI = (1.0001032, 1.000466); *p* = 1.41 × 10^−3^], simple mode [OR = 1.000446; 95% CI = (1.0001116, 1.000781); *p* = 1.04 × 10^−2^], and weighted mode [OR = 1.000285; 95% CI = (1.0001032, 1.000466); *p* = 2.29 × 10^−3^] showed similar results.

**TABLE 1:** Mendelian randomization analysis results of causal effects from RA to mechanical complication of prosthesis.

Exposure	Method	SNP (n)	OR	95% CI OR	*p*-value
RA	Weighted median	23	1.000285	(1.0001032, 1.000466)	1.41 × 10^−3^
RA	Inverse variance weighted	23	1.000255	(1.0001035, 1.000406)	9.69 × 10^−4^
RA	Simple mode	23	1.000446	(1.0001116, 1.000781)	1.04 × 10^−2^
RA	Weighted mode	23	1.000285	(1.0001032, 1.000466)	2.29 × 10^−3^

RA: Rheumatoid arthritis; MR: Mendelian randomization; SNP: single nucleotide polymorphism; β: The effect of the effect allele; and CI: Confidence interval.

### Sensitivity Analysis

**FIGURE 3:**
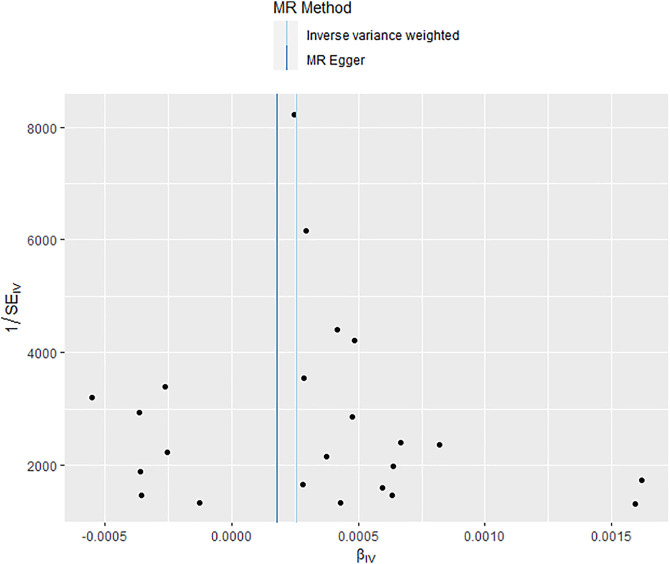
Funnel plot showed no significant heterogeneity among single nucleotide polymorphisms (SNPs).

**FIGURE 4:**
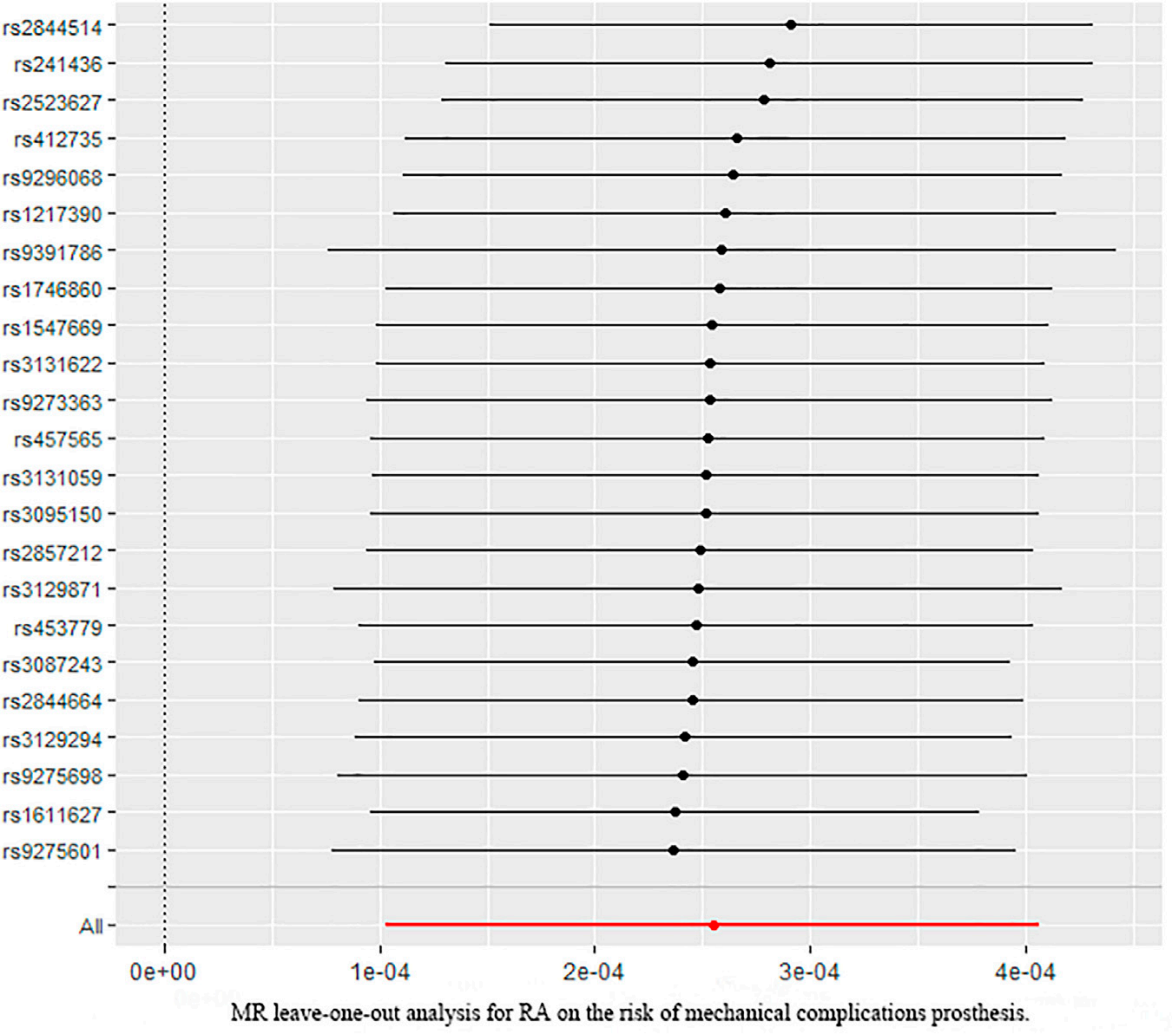
Leave-one-out analysis plots for RA on the risk of mechanical complications of prosthesis.

Additional sensitivity analysis was performed to identify the plausible biases caused by the IVW method. To evaluate heterogeneity, the Cochran’s Q test was performed, and the result showed no significant heterogeneity among SNPs (Q = 31.591; *p* = 0.085) (Figure 3). The MR-Egger intercept was applied to evaluate the directional pleiotropy, with the results showing no directional pleiotropy (MR-Egger intercept = 3.053 × 10^−5^; SE = 5.354 × 10^−5^; *p* = 0.575). Furthermore, the MR-PRESSO test was used to validate the absence of outliers and potential pleiotropy. Finally, the leave-one-out sensitivity test was performed, and the results showed that the causal effect of RA on mechanical complications of prosthesis was not significantly fluctuated with any single SNP leave-out (Figure 4), suggesting the stability and reliability of the MR analysis results.

## Discussion

With the advent of an aging society, arthroplasty surgery is performed on more patients. According to Steven et al., the number of total hip arthroplasties in the United States is estimated to be 572,000, while the number of total knee arthroplasties is estimated to reach 3.48 million by 2030 (Kurtz et al., 2007). Total knee arthroplasty and total hip arthroplasty are two common surgical strategies in the elderly, both aiming to provide pain relief caused by various articular diseases and restore joint function to meet the needs of daily life. With the growing number of patients receiving arthroplasty, knowledge on improving surgical techniques and patients’ satisfaction is of great significance. Although a great deal of attention has centered on surgical techniques such as artificial intelligence–designed patient-specific prosthesis (Spencer-Gardner et al., 2016) and computer navigation arthroplasty (Mathew et al., 2020), the postoperative complications should not be neglected as it is closely related to patient’s satisfaction. Complications of arthroplasty include aseptic loosening, periprosthetic joint infection, and pain, to name a few. In addition, mechanical complications including wearing, dislocation, and prosthesis fracture are common after surgery and may result in aseptic loosening. Thus, it is urgent to clarify the factors related to mechanical complications.

Rheumatoid arthritis (RA) is an autoimmune disease, which potentially results in joint deformities at the end stage. With this, several patients would opt to undergo arthroplasty as a last resort to recover joint function. Due to the systemic nature of RA, its postoperative complications differ from other articular diseases. Christoph Bohler et al. have suggested that compared to patients with osteoarthritis (a disease without systemic inflammation) who receive total knee arthroplasty, RA potentially increases the risk of aseptic loosening and the number of patients who require revision surgery (Böhler et al., 2020). Grzelecki et al. have reported that compared with non-RA patients, the clinical outcomes after revision surgery were similar with that of RA patients (Grzelecki et al., 2019). This indicates that the relationship between RA and complications are not yet fully established. Furthermore, previous studies were mostly retrospective studies where confounders and biases could not be eliminated. Therefore, the causal effects of RA on postoperative complications are still uncertain.

As far as the authors are aware, this was the first study to use the Mendelian randomization to explore the causal effects of RA on mechanical complications of prosthesis after arthroplasty. Mendelian randomization was utilized to investigate the causal effects by using SNPs as IVs and has been widely used in epidemiological studies of various diseases. The present study showed that RA potentially increases the odds of complications of prosthesis [OR = 1.000255; 95% CI = (1.0001035, 1.000406); *p* = 9.69 × 10^−4^], and this result was considered stable and reliable upon verification by sensitivity analysis. Based on this result, greater attention should be given to RA patients who are going to receive arthroplasty. In addition, the treatment of RA and the specific-type prosthesis should be taken into consideration for these patients.

A number of limitations are present in this study. First, the present study only focused on the causal effects of RA on mechanical complication of prosthesis; however, it is still unclear whether the course and disease activity of RA, social health determinants such as mental health and economic circumstances of RA patients have a role in this relationship. Second, this study was based on European ancestry and such a causal effect might be different in other races. To increase the reliability of this result, further MR analysis based on different ancestries should be performed. Third, the power of MR analysis could not be calculated since the SD of the GWAS analysis of the RA was not included in the study. Finally, weak instrument bias might arise in MR and result in weak relationships.

In summary, the present study performed a two-sample MR analysis for the first time to investigate the causal effect of RA on mechanical complications of prosthesis. Results of this study showed that RA potentially increases the risk of the mechanical complications of prosthesis after arthroplasty.

## Data Availability

The original contributions presented in the study are included in the article/Supplementary Material, further inquiries can be directed to the corresponding author.
