# Supplementary material for: The Causal Relationship Between Rheumatoid Arthritis and Mechanical Complications of Prosthesis After Arthroplasty: A Two-Sample Mendelian Randomization Study
Source: Front Genet. 2022 Apr 5;13:822448. doi: 10.3389/fgene.2022.822448 (PMC9016187; doi:10.3389/fgene.2022.822448)
Supplement: Supplementary file 1 [file Table1.DOCX]

**Supplementary Table 1.**

The calculation processes of Mendelian randomization of this study were listed as followings:

| 1 | Library (TwoSampleMR) |
| --- | --- |
| 2 | bmi_exp <- extract_instruments ( |
| 3 | outcomes='ieu-a-834', |
| 4 | clump=TRUE, r2=0.1, |
| 5 | kb=5000, access_token = NULL |
| 6 | ) |
| 7 | dim(bmi_exp) |
|  |  |
| 8 | t2d_out <- extract_outcome_data( |
| 9 | snps=bmi_exp$SNP, |
| 10 | outcomes='ukb-b-14753', |
| 11 | proxies = FALSE, |
| 12 | maf_threshold = 0.01, |
| 13 | access_token = NULL |
| 14 | ) |
| 15 | dim(t2d_out) |
|  |  |
| 16 | mydata <- harmonise_data( |
| 18 | exposure_dat=bmi_exp, |
| 19 | outcome_dat=t2d_out, |
| 20 | action= 2 |
| 21 | ) |
| 22 | res <- mr(mydata) |
| 23 | res |
| 24 | generate_odds_ratios(res) |
|  |  |
| 25 | het <- mr_heterogeneity(mydata) |
| 26 | het |
|  |  |
| 27 | pleio <- mr_pleiotropy_test(mydata) |
| 28 | pleio |
|  |  |
| 29 | single <- mr_leaveoneout(mydata) |
| 30 | mr_leaveoneout_plot(single) |
|  |  |
| 31 | mr_scatter_plot(res,mydata) |
|  |  |
| 32 | res_single <- mr_singlesnp(mydata) |
| 33 | mr_forest_plot(res_single) |
|  |  |
| 34 | mr_funnel_plot(res_single) |
