# Supplementary material for: The Causal Relationship Between Rheumatoid Arthritis and Mechanical Complications of Prosthesis After Arthroplasty: A Two-Sample Mendelian Randomization Study
Source: Front Genet. 2022 Apr 5;13:822448. doi: 10.3389/fgene.2022.822448 (PMC9016187; doi:10.3389/fgene.2022.822448)
Supplement: Supplementary file 2 [file Table2.DOCX]

**Supplementary file 2.**

**Supplementary Table 1.** The single nucleotide polymorphism selected for rheumatoid arthritis to perform Mendelian randomization analysis.

| SNP | EA | Chromosome | β | SE | *P*-value |
| --- | --- | --- | --- | --- | --- |
| rs11102712 | T | 1 | 0.307485 | 0.0393242 | 3.95458e-15 |
| rs2476601 | G | 1 | -0.662688 | 0.0381065 | 6.22014e-71 |
| rs1217390 | T | 1 | 0.162519 | 0.0268777 | 9.20704e-10 |
| rs1746860 | C | 1 | -0.150823 | 0.0237476 | 4.68587e-09 |
| rs1578712 | G | 1 | -0.246860 | 0.0377883 | 1.93232e-10 |
| rs3087243 | A | 2 | -0.139262 | 0.0234742 | 2.24006e-08 |
| rs10040327 | A | 5 | -0.287682 | 0.0440339 | 6.64967e-11 |
| rs2844664 | T | 6 | 0.262364 | 0.0254335 | 3.63664e-24 |
| rs7769073 | A | 6 | -0.478036 | 0.0816754 | 6.36034e-09 |
| rs3094165 | G | 6 | 0.162519 | 0.0240274 | 2.30988e-10 |
| rs9261858 | A | 6 | 0.207014 | 0.0332309 | 2.29525e-10 |
| rs2523841 | A | 6 | -0.261365 | 0.0362690 | 1.38580e-13 |
| rs3129871 | C | 6 | 0.673345 | 0.0247867 | 1.53815e-152 |
| rs6920220 | A | 6 | 0.198851 | 0.0293062 | 2.49345e-12 |
| rs2249099 | A | 6 | 0.223144 | 0.0386950 | 4.01819e-09 |
| rs2429657 | G | 6 | -0.248461 | 0.0292795 | 1.34834e-17 |
| rs2857096 | C | 6 | 0.262364 | 0.0274991 | 3.57767e-20 |
| rs1063478 | T | 6 | -0.248461 | 0.0423431 | 5.39722e-09 |
| rs3873444 | T | 6 | -0.673345 | 0.0446949 | 3.36357e-54 |
| rs12204421 | G | 6 | -0.210721 | 0.0281995 | 1.03872e-13 |
| rs1547669 | G | 6 | 0.173953 | 0.0234988 | 1.75428e-13 |
| rs2844514 | T | 6 | -0.342490 | 0.0249950 | 5.99101e-41 |
| rs2523679 | T | 6 | 0.488580 | 0.0453666 | 2.71331e-28 |
| rs424232 | T | 6 | -0.371064 | 0.0296102 | 8.28514e-42 |
| rs2621330 | T | 6 | -0.494296 | 0.0625312 | 1.21703e-14 |
| rs4148876 | A | 6 | 0.398776 | 0.0461978 | 2.26360e-18 |
| rs2857212 | G | 6 | 0.215111 | 0.0247066 | 1.01859e-17 |
| rs3097646 | C | 6 | 0.774727 | 0.0411398 | 1.21703e-77 |
| rs380468 | G | 6 | -0.415515 | 0.0309594 | 1.32770e-45 |
| rs1042448 | A | 6 | -0.356675 | 0.0291863 | 2.29879e-39 |
| rs6901724 | C | 6 | -0.287682 | 0.0409037 | 7.59102e-13 |
| rs3131622 | G | 6 | 0.139762 | 0.0221968 | 3.90086e-09 |
| rs3025654 | T | 6 | 0.548121 | 0.0704145 | 9.01364e-15 |
| rs3129294 | C | 6 | -0.261365 | 0.0265280 | 8.84097e-25 |
| rs9264374 | T | 6 | 0.210721 | 0.0315342 | 1.26794e-12 |
| rs521828 | T | 6 | -0.478036 | 0.0247066 | 5.32844e-69 |
| rs1800454 | T | 6 | 0.476234 | 0.0363919 | 3.09172e-41 |
| rs9461839 | C | 6 | -0.385662 | 0.0560866 | 1.50280e-12 |
| rs2296330 | A | 6 | 0.262364 | 0.0274991 | 1.10459e-20 |
| rs497239 | C | 6 | -0.579818 | 0.0456756 | 5.60790e-41 |
| rs169503 | T | 6 | -0.544727 | 0.0873179 | 1.95718e-10 |
| rs10484560 | A | 6 | 0.231112 | 0.0383880 | 7.44646e-09 |
| rs7745656 | T | 6 | -0.544727 | 0.0264134 | 2.88071e-80 |
| rs3909134 | G | 6 | 0.530628 | 0.0838021 | 1.57438e-10 |
| rs530878 | G | 6 | 0.198851 | 0.0312761 | 1.57438e-10 |
| rs1624944 | C | 6 | -0.157004 | 0.0282529 | 1.65852e-08 |
| rs2523619 | C | 6 | -0.314711 | 0.0312761 | 2.60976e-24 |
| rs9277771 | A | 6 | 0.364643 | 0.0319294 | 5.72400e-31 |
| rs9391786 | G | 6 | 0.916291 | 0.0234390 | 1.00000e-200 |
| rs9273363 | A | 6 | 0.405465 | 0.0272367 | 2.99640e-53 |
| rs6913635 | G | 6 | 0.392042 | 0.0499771 | 1.06782e-14 |
| rs457565 | A | 6 | -0.235722 | 0.0258552 | 1.17220e-19 |
| rs999265 | C | 6 | -0.186330 | 0.0307724 | 9.39356e-09 |
| rs1419675 | T | 6 | 0.210721 | 0.0281995 | 5.42875e-14 |
| rs13201129 | C | 6 | 0.182322 | 0.0297957 | 2.32060e-09 |
| rs9296011 | G | 6 | 0.774727 | 0.0598158 | 1.21227e-37 |
| rs9404942 | T | 6 | -0.385662 | 0.0712172 | 3.64813e-08 |
| rs17576984 | T | 6 | -0.579818 | 0.0456756 | 9.40156e-37 |
| rs9268498 | C | 6 | -0.400478 | 0.0458126 | 8.78618e-20 |
| rs2074488 | T | 6 | 0.444686 | 0.0342824 | 1.05609e-40 |
| rs3892710 | T | 6 | 0.182322 | 0.0317965 | 7.66637e-09 |
| rs9296081 | G | 6 | -0.462035 | 0.0769070 | 7.60466e-10 |
| rs4711363 | G | 6 | -0.174353 | 0.0304052 | 1.75829e-09 |
| rs9275698 | G | 6 | -0.478036 | 0.0247066 | 3.06902e-78 |
| rs2975046 | G | 6 | -0.235722 | 0.0323346 | 6.16595e-13 |
| rs9272535 | A | 6 | -0.579818 | 0.0273586 | 2.05069e-93 |
| rs10484564 | C | 6 | -0.430783 | 0.0472302 | 2.74347e-19 |
| rs412735 | A | 6 | 0.262364 | 0.0314369 | 1.95434e-16 |
| rs11757182 | C | 6 | -0.478036 | 0.0865346 | 2.85319e-08 |
| rs4711211 | G | 6 | 0.329304 | 0.0274568 | 3.38298e-33 |
| rs241436 | G | 6 | -0.356675 | 0.0253501 | 9.76338e-46 |
| rs3819721 | A | 6 | 0.657520 | 0.0277127 | 2.46037e-126 |
| rs154977 | G | 6 | 0.415515 | 0.0309594 | 1.73261e-38 |
| rs2523627 | T | 6 | 0.314711 | 0.0243138 | 2.89401e-37 |
| rs3806155 | T | 6 | 1.360980 | 0.0711207 | 2.67732e-81 |
| rs9275601 | T | 6 | -0.446287 | 0.0277127 | 2.47970e-55 |
| rs2071465 | G | 6 | 0.270027 | 0.0252398 | 5.13098e-27 |
| rs9296068 | G | 6 | -0.210721 | 0.0252158 | 1.44212e-15 |
| rs2535260 | G | 6 | -0.210721 | 0.0315342 | 6.06038e-12 |
| rs6922431 | C | 6 | -0.446287 | 0.0515910 | 2.55564e-18 |
| rs2856683 | G | 6 | 0.425268 | 0.0267017 | 2.80027e-54 |
| rs2239800 | G | 6 | -0.342490 | 0.0465106 | 5.30884e-14 |
| rs3131059 | C | 6 | 0.157004 | 0.0261873 | 5.01510e-10 |
| rs3095150 | T | 6 | 0.174353 | 0.0271964 | 1.02204e-10 |
| rs9394026 | A | 6 | 0.198851 | 0.0293062 | 4.35111e-12 |
| rs1265093 | A | 6 | 0.270027 | 0.0252398 | 6.02560e-26 |
| rs12202667 | C | 6 | -0.400478 | 0.0381459 | 6.14752e-27 |
| rs474534 | G | 6 | 0.292670 | 0.0381459 | 9.44061e-15 |
| rs9275602 | A | 6 | 0.371564 | 0.0333598 | 2.21411e-30 |
| rs6899309 | C | 6 | -0.527633 | 0.0608191 | 1.41449e-18 |
| rs7772982 | C | 6 | 0.173953 | 0.0300467 | 1.42978e-08 |
| rs2244579 | C | 6 | -0.248461 | 0.0292795 | 1.15718e-17 |
| rs2844463 | A | 6 | -0.385662 | 0.0375828 | 7.93049e-24 |
| rs9469079 | T | 6 | -0.415515 | 0.0465106 | 6.65426e-19 |
| rs17421624 | C | 6 | 0.559616 | 0.0247301 | 3.23594e-108 |
| rs11752428 | C | 6 | -0.733969 | 0.0687917 | 7.34345e-26 |
| rs2516713 | A | 6 | -0.198451 | 0.0311486 | 1.92522e-09 |
| rs9295912 | C | 6 | -0.223144 | 0.0255315 | 1.21423e-16 |
| rs915654 | A | 6 | 0.336472 | 0.0255315 | 2.54156e-38 |
| rs3093662 | G | 6 | -0.446287 | 0.0515910 | 2.40049e-19 |
| rs2269475 | T | 6 | 0.364643 | 0.0335913 | 4.36114e-26 |
| rs9262602 | G | 6 | 0.277632 | 0.0348407 | 6.03393e-16 |
| rs707937 | G | 6 | -0.198451 | 0.0311486 | 1.72223e-10 |
| rs6906128 | C | 6 | -0.510826 | 0.0255315 | 1.22999e-79 |
| rs9268145 | G | 6 | 1.057790 | 0.0265972 | 1.00000e-200 |
| rs16870126 | G | 6 | -0.755023 | 0.0593424 | 4.09449e-34 |
| rs453779 | G | 6 | 0.301105 | 0.0276055 | 4.56142e-34 |
| rs1611627 | C | 6 | -0.186330 | 0.0246072 | 9.51262e-15 |
| rs9262544 | A | 6 | -0.371064 | 0.0404605 | 6.95344e-19 |
| rs8192575 | G | 6 | 1.088560 | 0.0454141 | 4.63447e-126 |
| rs3104402 | G | 6 | 0.544727 | 0.0481100 | 2.64850e-27 |
| rs154973 | T | 6 | -0.415515 | 0.0500293 | 3.13473e-16 |
| rs9391754 | A | 6 | -0.462035 | 0.0487386 | 4.01051e-21 |
| rs210194 | G | 6 | -0.261365 | 0.0428921 | 5.75732e-09 |
| rs4810485 | G | 20 | 0.162519 | 0.0300467 | 5.69023e-09 |
