# Supplementary material for: The Causal Relationship Between Rheumatoid Arthritis and Mechanical Complications of Prosthesis After Arthroplasty: A Two-Sample Mendelian Randomization Study
Source: Front Genet. 2022 Apr 5;13:822448. doi: 10.3389/fgene.2022.822448 (PMC9016187; doi:10.3389/fgene.2022.822448)
Supplement: Supplementary file 3 [file Table3.DOCX]

1. The Two-sample Mendelian randomization based on GWAS summary data published by Okada et al. at 2014(Okada et al. 2014), the MR results were listed in the following table.

| **Exposure** | **Method** | **SNP(n)** | **OR** | **95%CI OR** | ***P*-value** |
| --- | --- | --- | --- | --- | --- |
| RA | Weighted median | 62 | 1.000308 | (1.0000797, 1.000537) | 0.00822 |
| RA | Inverse variance weighted | 62 | 1.000254 | (1.000108, 1.000401) | 6.58×10^-4^ |
| RA | Simple mode | 62 | 1.000134 | (0.9996984, 1.000570) | 0.548 |
| RA | Weighted mode | 62 | 1.000326 | (1.0000962, 1.000556) | 0.00721 |
| RA | MR Egger | 62 | 1.000353 | (1.0000900, 1.000616) | 0.0108 |

RA: Rheumatoid arthritis; MR: Mendelian randomization; SNP: single nucleotide polymorphism; β: The effect of the effect allele; CI: Confidence interval.

2. The Two-sample Mendelian randomization based on GWAS summary data published by Steve Eyre et al. at 2012(Eyre et al. 2012), the MR results were listed in the following table.

| **Exposure** | **Method** | **SNP(n)** | **OR** | **95%CI OR** | ***P*-value** |
| --- | --- | --- | --- | --- | --- |
| RA | Weighted median | 27 | 1.000336 | (1.0001141, 1.000557) | 2.98×10^-3^ |
| RA | Inverse variance weighted | 27 | 1.000330 | (1.000167, 1.000493) | 7.15×10^-5^ |
| RA | Simple mode | 27 | 1.000443 | (1.0000812, 1.000805) | 2.39×10^-2^ |
| RA | Weighted mode | 27 | 1.000339 | (1.0001214, 1.000558) | 5.19×10^-3^ |
| RA | MR Egger | 27 | 1.000264 | (0.9999704 1.000558) | 9.02×10^-2^ |

RA: Rheumatoid arthritis; MR: Mendelian randomization; SNP: single nucleotide polymorphism; β: The effect of the effect allele; CI: Confidence interval.

Reference

Eyre, Steve, John Bowes, Dorothée Diogo, Annette Lee, Anne Barton, Paul Martin, Alexandra Zhernakova, et al. 2012. “High-Density Genetic Mapping Identifies New Susceptibility Loci for Rheumatoid Arthritis.” *Nature Genetics* 44 (12): 1336–40. https://doi.org/10.1038/ng.2462.

Okada, Yukinori, Di Wu, Gosia Trynka, Towfique Raj, Chikashi Terao, Katsunori Ikari, Yuta Kochi, et al. 2014. “Genetics of Rheumatoid Arthritis Contributes to Biology and Drug Discovery.” *Nature* 506 (7488): 376–81. https://doi.org/10.1038/nature12873.
